# Incidence and root causes of delays in emergency orthopaedic procedures: a single-centre experience of 36,017 consecutive cases over seven years

**DOI:** 10.1186/s13037-018-0149-1

**Published:** 2018-01-11

**Authors:** Ulla Caesar, Jon Karlsson, Elisabeth Hansson

**Affiliations:** 1 Sahlgrenska Academy, Institute of Clinical Sciences, Department of Orthopaedics, University of Gothenburg Sweden, Gothenburg, Sweden; 2 Sahlgrenska Academy, Institute of Health and Care Sciences, University of Gothenburg Sweden, Gothenburg, Sweden; 3 Department of Orthopaedics, Sahlgrenska University Hospital, Gothenburg, Sweden

**Keywords:** Appointments and schedules, Operating rooms/organisation and administration, Waiting lists, Cancellation, Orthopaedic surgery, Emergency delays, Perioperative nursing

## Abstract

**Background:**

Emergency surgery is unplanned by definition and patients are scheduled for surgery with minimal preparation. Some patients who have sustained emergency orthopaedic trauma or other conditions must be operated on immediately or within a few hours, while others can wait until the hospital’s resources permit and/or the patients’ health status has been optimised as needed. This may affect the prioritisation procedures for both emergency and elective surgery and might result in waiting lists, not only for planned procedures but also for emergencies.

**Method:**

The main purpose of this retrospective, observational, single-centre study was to evaluate and describe for the number and reasons of delays, as well as waiting times in emergency orthopaedic surgery using data derived from the hospital’s records and registers. All the emergency patients scheduled for emergency surgery whose procedures were rescheduled and delayed between 1 January 2007 and 31 December 2013 were studied.

**Result:**

We found that 24% (8474) of the 36,017 patients scheduled for emergency surgeries were delayed and rescheduled at least once, some several times. Eighty per cent of these delays were due to organisational causes. Twenty-one per cent of all the delayed patients had surgery within 24 h, whilst 41% waited for more than 24 h, up to 3 days.

**Conclusion:**

A large number of the clinic’s emergency orthopaedic procedures were rescheduled and delayed and the majority of the delays were related to organisational reasons. The results can be interpreted in two ways; first, organisational reasons are avoidable and the potential for improvement is great and, secondly and most importantly, the delays might negatively affect patient outcomes.

## Background

High availability and good quality are regarded as important goals in the Swedish health-care system (80% publicly funded) [1], which aims to provide care on equal terms to all citizens [2]. Delivering care equally and efficiently with high quality is a challenge. One of the areas affected by these challenges is surgery. Emergency surgery is unplanned by definition and patients are scheduled with minimal forward planning. As a result, they often have to be fitted into a surgery schedule as competition to the electives, when operating room (OR) space is limited. They may otherwise be included as a more highly prioritised emergency disruption to the elective OR list [3–11]. When operations are rescheduled and the waiting time is prolonged, a deterioration in the patients’ health may result in unnecessary suffering [12–16], which may in turn cause postponed or poorer recovery and possibly inferior outcomes [17, 18]. Moreover, studies of cancellations of and delays in surgical procedures reveal the inefficient use of hospital resources, as well as a loss of hospital revenues [19–23].

Despite improvements in technical and hospital resources over the last few decades, surgical delays still occur every day in orthopaedic departments [24–26]. These delays could be explained by the high demands related to emergency orthopaedic admissions covering a wide spectrum of injuries that often require surgical management [27]. True orthopaedic emergencies that require immediate surgery are conditions such as acute compartment syndromes and fractures or dislocations associated with vascular injury. Some of the other emergencies can wait without harm, but they have to be managed as soon as the patient’s status has been optimised and when the hospital’s resources permit [27]. Taken together, emergencies must be considered in terms of the seriousness of the injury, the patient’s health status and access to the OR. Moreover, planned surgeries wait for months to be performed. All this requires the prioritisation of the included patients and will in itself create waiting lists for both emergency and elective surgery. In addition, some emergencies enter the OR as competitors when elective surgeries have to be rescheduled [24].

Even though a common reason for cancelling surgery is a prioritised emergency, a growing body of research has focused on delays to and cancellations in elective surgical procedures [3–10, 24, 28]. Moreover, studies have shown a decrease in delayed surgery, when the causes of the delays have been identified [29, 30]. Irrespective of the reason for delays to emergency surgeries, they lead to concerns for the patients and major organisational problems for the clinics. Problems such as extended waiting lists and an overbooked OR, a shortage of hospital beds and cancellations of either elective or emergency surgery at the end of the day are commonly mentioned. Magnusson et al. [31] claimed that a delay might contribute to unnecessary distress and might also lead to a loss of confidence in the hospital. It has also been revealed that feelings of insecurity might lead to increased pain which can in turn lead to a prolonged hospital stay [13, 15, 32]. The main purpose of the current study was to evaluate and describe the number of and reasons for delays, as well waiting time in emergency orthopaedic procedures, at a clinic routinely performing both acute and elective orthopaedic surgery.

## Method

The study was a descriptive single-centre study with the retrospective inclusion of prospective, observational data derived from the hospital’s records and registers. The study population comprised all the patients scheduled for emergency orthopaedic surgery, from 1 January 2007 to 31 December 2013 at a university hospital with an annual production of approximately 9500 procedures comprising 46% planned and 54% acute surgical procedures. The orthopaedic clinic is organised into specialised teams focusing on trauma, joint replacement, arthroscopic, foot & ankle, tumour, paediatric orthopaedic and spine surgery.

The included cases were patients scheduled for emergency procedures who were then delayed. The collected data included age, gender, diagnosis, reason for delay, time of delay and length of time until surgery was performed. The age of the patients ranged between three and 107 years and 54% of patients were women. During the study period, a total of 36,017 patients were on the surgical emergency waiting list.

The patients entered the hospital from the emergency room (ER), the non-urgent surgery-consulting centre, or from a referral by a physician outside the hospital. All the patients underwent a medical examination and, in several cases, an imaging examination (radiographs, ultrasonography or magnetic resonance), before a decision to perform emergency surgery was made.

At the clinic, three emergency waiting lists ran in parallel each weekday, with three dedicated ORs on weekdays and two at weekends. One OR was specified for hip fracture patients and two for general orthopaedic trauma and “home pathway” patients. We identified patients from all these lists. The “home pathway” patients required surgery but could not be scheduled within 24 h after admission. These patients could be placed in plaster of Paris, a bandage or a sling before they were discharged to their homes to wait for surgery.

The OR schedule was based on priorities and decisions made by the surgeons and on the hospital’s working method, which are as follows.

1. A hip fracture is planned to undergo surgery within 24 h.
2. The emergency in-hospital patients waiting on the ward are planned to undergo surgery within 24 h.
3. “Home pathway” surgery

After the decision, a notification was sent to a co-ordinator who booked the appointment for the surgical procedure. This meant that patient data were entered into the planning system and a file with a patient ID was opened in the electronic planning system (Operätt). In this system, co-ordinators, surgeons and nurses registered data.

To confirm the daily structure and order of priority on the three emergency waiting lists, a regular morning meeting was held at the OR Department. There and then, a senior surgeon prioritised the daily schedule for all the surgery procedures. In a few cases, the senior surgeon did not find any indication for surgery and the procedure was cancelled. After the prioritisation was complete, the co-ordinator contacted the wards to confirm the patients waiting for surgery in hospital. Moreover, phone calls were made to those waiting at home, to inform them of either a delay or a definite time for surgery.

The IT tool, Qlick View (QV), was used as a database and made it possible to identify, calculate and present quality measurements of all activities associated with the emergency surgery waiting list. Qlick View also enabled the identification of all cancellations made in the planning system. The planning system was updated every day and validated every month.

Delays and cancellations were entered into the planning system under one of 47 possible codes and they were merged into three categories; organisational, medical and patient-related reasons. The organisational reasons were due to:

- An emergency case with higher priority
- The unexpected prolongation of on-going surgery
- Incomplete pre-operative preparations and need for further patient evaluation
- Changed or missing indications for surgery
- Shortage of hospital beds on the wards or at the Intensive care unit (ICU)
- Shortage of staff in the OR, ICU, ward and/or surgeons and anaesthesiologists

Moreover, some of the organisational reasons included the patient being referred to another hospital or another surgical team. The medical reasons included disorders that made the patient inappropriate at the planned time point or totally inoperable, or else that the patient had an on-going infection. The patient-related category was due to the patients’ personal wishes to have surgery performed on a later occasion, the patients not showing up at the appointed time or the patients dying or refusing surgery.

The data were managed using IBM SPSS Statistics (Version 21). Descriptive data were presented in absolute and relative numbers, median and range values. Graphics were illustrated using Microsoft Excel (Version 2013).

## Result

**Fig. 1:**
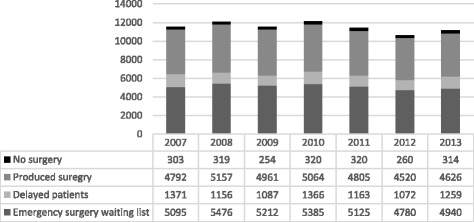
Produced and delayed emergency surgery 2007–2013. *Emergency surgery waiting list*; every new patient is entered into an electronic surgical planning system as a file with a unique patient ID. The patient remains in the planning system until the operation is completed, transferred to another care-giver or the patient did not require surgery. *Produced surgery*; all the patients who underwent surgery at the current clinic. *Delayed patients*; all the patients that were delayed. *No surgery*; surgery on the waiting list that did not undergo surgery at the current clinic

Of all 36,017 patients who were scheduled for emergency surgery, (6604/36,017) 18% had their procedure cancelled once, 4% (1490/36,017) twice, 1% (289/36,017) three times, < 1% (58/36,017) four times and < 1% (33/36,017) more than four times. This adds up to a total number of 10,873 delays for the 8474 actual patients. The produced surgeries totalled 33,925 and 2090 cases on the waiting list did not undergo surgery at the current clinic. The proportions of delays and delayed patients did not change across the 7 years that were studied (Fig. 1).

**Fig. 2:**
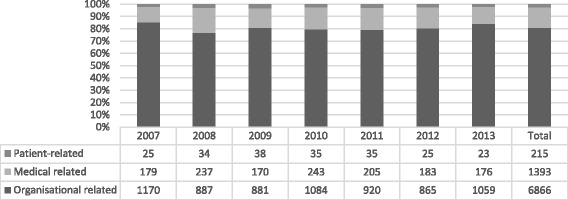
Reasons for all delayed patients’ emergency surgery 2007–2013

The two most frequent reasons within the organisational group were that another emergency surgery was given higher priority and the unexpected prolongation of on-going surgery. Missing OR equipment was the least frequent reason. The organisational reasons amounted to 81% (6865/8474) and, among them, 71% (4875/6865) were caused by a higher priority emergency case (Fig. 2). Together, the medical and the patient-related causes had fewer delays than the organisational causes. Sixteen per cent (1393/8474) of the delays were due to medical reasons and 3% (215/8474) were due to patient-related reasons (Fig. 2). The reasons for cancellation of emergency surgery were proportionally the same also when cancellations occurred several times.

**Fig. 3:**
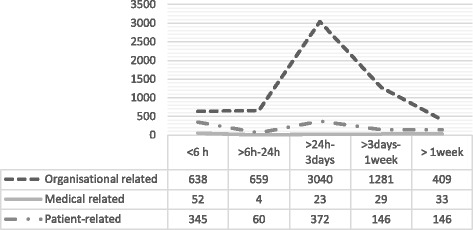
Waiting time for surgery for all delayed emergency patients by reasons 2007–2013

Of all the delayed emergencies, 21% (1035 + 721/8474) underwent surgery within 24 h. The highest number, 41% (3438/ 8474) waited for more than 24 h and up to 3 days, while 17% (1458/8474) waited from 3 days to 1 week or even more than 1 week (Fig. 3). The group that waited for more than 24 h up to 3 days decreased during the 7 years that were studied, while those who waited more than 1 week increased. Fifteen per cent (1237/8474) of all delays had no registered waiting time (Table 1).

**Table 1:** Waiting time to surgery for all delayed emergency patients 2007–2013

YEAR	< 6 h	> 6 h–24 h	> 24 h-3 days	> 3 days-1 week	> 1 week	missing	Total
2007	154	100	699	191	51		1195
2008	160	99	500	167	49		975
2009	121	89	478	176	65		929
2010	159	104	524	292	103		1182
2011	159	111	428	191	85		974
2012	138	120	387	192	85		922
2013	144	98	422	249	147		1060
Missing						1237	1237
Total	1035	721	3438	1458	585	1237	8474

## Discussion

The most important finding in the present study was the high frequency of rescheduled emergency orthopaedic surgery. Of all the patients scheduled for emergency surgery, 24% (8474/36,017) had their surgical procedure delayed at least once and some several times. Another important finding was that the proportions of delays did not change but were instead fairly constant, over the 7 years that were studied. In Sweden, the large university hospitals tend to have the longest waiting times for planned orthopaedic procedures [33]. Schuster et al. [34] have observed a similar situation in terms of waiting times at German university hospitals. It is important to acknowledge that university hospitals commonly deal with an increased volume of moderate to complex care patients transferred from smaller hospital. Consequently, the number of emergency patients increases and the situation with overbooked ORs becomes more complex, consistent with extended waiting lists and increasing waiting times.

The present study revealed a variety of reasons for the delays. When grouping, almost 80% were due to organisational reasons. The large number of delays in this group came from emergencies having an even higher priority. Several studies [10, 35, 36] have reported that emergencies are often the reason for cancellations of elective procedures. Moreover, the review by Cardoon et al. [24] observed that most of the research on the planning and scheduling of surgery focused on elective patients, while major organisational shortages were caused by the arrival of emergency patients. Moreover, Cosgrove et al. [29] and Leppaniemi and Jousela [30] have described methods for prioritising surgery, based on clinical outcomes and existing clinical guidelines. These findings demonstrate that hospitals will be better prepared to avoid delays when the reasons for delays have been clarified.

The second most frequent delay in the present study was related to medical reasons and accounted for 17% of all the delays. Studies [37, 38] have shown that medical reasons are not the most common cause of emergency-related delays. The medical delays are often due to respiratory and/or vascular complications. These and many of the other medically related reasons are unforeseeable and unavoidable and hospitals have few opportunities to influence these types of delay. Some of the delays might be helped by improving the organisation of the preoperative emergency assessment. Several studies have shown fewer delays, with a reduction of 1–8%, when preoperative routines for consulting and assessment have been improved [39].

One finding in the present study was that almost 70% of the delayed emergencies waited for more than 24 h up to 1 week before surgery. The acute emergencies which waited less than 6 hours (14%) might be regarded as not constituting a delay. It appears realistic to wait 6 hours, but the organisation of the OR scheduling is still disrupted, when patients are rescheduled. Emergency care in Sweden is not tied to the health-care guarantee. The prioritisation is determined by the urgency of the medical needs. Patients who have become acutely ill or injured will receive treatment as quickly as possible. However, the National Board of Health and Welfare [40] stepped up the goal in connection with hip-fracture care, stating that surgery should take place on the day of admission or within 24 h after admission to hospital. Taken as a whole, this results in the hospital’s part of prioritisation becoming problematic and recommendations for cut-off times may have both positive and negative effects on all the orthopaedic waiting lists and delays [41].

Another finding in the current study was that almost 6% (2090/36,017) of the patients on the waiting lists did not undergo surgery at the current clinic. Some of these patients could be explained by differences between the first prioritisation of surgery and the later prioritisation, made by the senior surgeon, who did not find any indication for surgery and the procedure was therefore cancelled. Moreover, the patients might not accept the long waiting time and accordingly search for care at another hospital.

When patients reschedule and cancel their surgery, it disrupts the operation planning procedure, especially when this is done at short notice. The present study revealed a low rate (2%) of patient-related reasons for delays in emergency surgery. It appears reasonable to suppose that patients do not reschedule acute surgery. On the other hand, almost 40% of the present clinic’s elective cancellations [28] were due to patient-related causes. Several other studies have also observed large numbers of patient-related reasons for cancelling elective surgery [42] and the rescheduling of surgery often leads to unutilised ORs [43].

To respond to the demand from all orthopaedic procedures and the disruptions among elective and emergency cases, the clinic organised the emergency cases into one OR dedicated to hip fractures and two other ORs for both the acute in-patients and the “home-care pathway” patients. The elective surgery was only performed on weekdays and had its own schedule and dedicated OR. Several studies have [44–47] reported that separating emergency from elective surgery reduces delays and cancellations. This was also the case in the present study, but it was not true in terms of the cancellations of emergency procedures; 71% (4875/6865) of the organisational delays were caused by an even more highly prioritised emergency case. We also found that approximately 80% of the present clinic’s delayed emergency procedures had organisational causes. In a previous study, Caesar et al. [28] revealed that 9% of the same clinic’s elective surgeries were cancelled due to organisational shortages. Taken together, these results illustrate that emergencies are more frequently delayed by an ineffectual organisation rather than planned surgery, which indicates that emergencies are more often delayed by other more highly prioritised emergencies than electives.

Another way to avoid over-booked ORs, as well as the shortage of ward space, was the clinic’s organisation using a “home pathway”, including patients waiting at home. The “home pathway” is a new concept within orthopaedics. The limited available literature [48, 49] indicates that home care is safe if the instructions to select patients, patient information, and contact with the hospital are appropriate and clearly clarified. The results of the current study demonstrated that the group that waited for more than 24 h up to 3 days decreased during the 7 years that were studied, while those who waited more than a week increased. This might indicate a value of the “home pathway”, as more patients waited at home and did not compete with other surgery, thereby allowing the present clinic to schedule surgery in the short term. In addition, this might have ensured the streamlining of the emergency waiting list and may be a more realistic approach to booking surgery and, in the longer term, lead to fewer delays and cancellations.

Surgical complications increase, the longer the waiting time to surgery [50]. Delays longer than 24 h have proved to be an important risk factor for wound infection in hip fractures. Moreover, older age in patients with hip fractures was a significant risk factor for mortality, upper urinary tract infections and pneumonia [51]. Likewise, if ankle fractures were delayed more than 48 h, the length of stay in hospital and the cost increased significantly. Ankle fractures in individuals over 70 years of age have been shown a high rate of complications, such as infection and delayed wound healing, and they therefore need to be prioritised [52]. Pettersson et al. [41] demonstrated that 20% of hip-fracture patients suffered a serious adverse event during their hospital stay and the risk of complications occurring increased linearly over time. Reducing delays in the preoperative care process of orthopaedic emergency surgery appears to reduce the risk of complications and serious adverse events in patients. One way might be to implement procedures for prioritisation based on evidence from research and clinics.

The “home pathway”, at least at the current clinic, is an unexplored pathway and a fairly new phenomenon in emergency care. The organisational outcomes and waiting times, as well as patients’ experiences of waiting at home for emergency orthopaedic surgery, are areas for further research.

### Limitations

There are several limitations to this study. First, as the data come from a single hospital, the results are difficult to generalise to other orthopaedic clinics, with different functional characteristics, such as size, services provided and case mix. Another limitation could be that different staff categories entered the data into the surgical planning system, Operätt. This could lead to the inconsistent grouping of the reasons for cancellations. Since there is both a continuous inflow and outflow from the current clinic’s waiting lists, the numbers given may vary. This makes it difficult to provide the precise numbers from one moment to another.

## Conclusion

Hospitals and clinics need to deal with the root causes of inefficiency and shortages in many ways. Clarifying the reasons for delays to orthopaedic procedures is the first and probably the most important step when it comes to dealing with the root causes and shortages at the present hospital, in order to decrease both elective and acute surgery delays and cancellations.

The large number of organisational delays in the present study is a major quality problem affecting the individual patient and the actual health-care organisation, as well as prolonging sick leave. While many of the organisational reasons are avoidable, some of them are still caused by factors that are outside the responsibility of the individual clinic or even the hospital. Many of the delays, such as the medical reasons, appear to be impossible to reduce or eliminate, but some might nonetheless be helped by improving the organisation of preoperative emergency assessments.
